# Bufotalin Suppresses Proliferation and Metastasis of Triple-Negative Breast Cancer Cells by Promoting Apoptosis and Inhibiting the STAT3/EMT Axis

**DOI:** 10.3390/molecules28196783

**Published:** 2023-09-23

**Authors:** So Jin Park, Hye Jin Jung

**Affiliations:** 1 Department of Life Science and Biochemical Engineering, Graduate School, Sun Moon University, Asan 31460, Republic of Korea; psj1867@naver.com; 2 Department of Pharmaceutical Engineering and Biotechnology, Sun Moon University, Asan 31460, Republic of Korea; 3 Genome-Based BioIT Convergence Institute, Sun Moon University, Asan 31460, Republic of Korea

**Keywords:** triple-negative breast cancer, bufotalin, apoptosis, epithelial-mesenchymal transition, signal transducer and activator of transcription 3

## Abstract

Triple-negative breast cancer (TNBC) is a highly aggressive type of breast cancer and has a poor prognosis. As standardized TNBC treatment regimens cause drug resistance and tumor recurrence, the development of new TNBC treatment strategies is urgently required. Bufotalin is a bufadienolide isolated from the skin and parotid venom glands of the toad *Bufo gargarizan*, and has several pharmacological properties, including antiviral, anti-inflammatory, and anticancer activities. However, the anticancer effect and underlying molecular mechanisms of action of bufotalin in TNBC have not been fully studied. In the current study, we investigated the effects of bufotalin on the growth and metastasis of MDA-MB-231 and HCC1937 TNBC cells. Bufotalin potently inhibited the proliferation of both TNBC cell lines by promoting cell cycle arrest and caspase-mediated apoptosis. Furthermore, bufotalin effectively suppressed the migration and invasion of both TNBC cell lines by regulating the expression of key epithelial-mesenchymal transition (EMT) biomarkers, matrix metalloproteinases (MMPs), and integrin α6. Notably, the anticancer effect of bufotalin in TNBC cells was associated with the downregulation of the signal transducer and activator of the transcription 3 (STAT3) signaling pathway. Collectively, our results suggest that the natural compound bufotalin may exert antiproliferative and antimetastatic activities in TNBC cells by modulating the apoptotic pathway and the STAT3/EMT axis.

## 1. Introduction

Breast cancer is the most commonly diagnosed disease in women worldwide, with an incidence of 2.3 million and 685,000 deaths reported in 2020 [1]. Breast cancer subtypes are generally classified into estrogen receptor positive (ER+), progesterone receptor positive (PR+), human epidermal growth factor receptor 2 positive (HER2+), and triple-negative [2,3]. Triple-negative breast cancer (TNBC) accounts for 15–20% of all breast cancer cases and is the breast cancer type that does not express HER2, ER, and PR [2,3]. TNBC has a higher rate of distant recurrence and a worse overall prognosis than HER2- or hormone receptor-positive breast cancer types [3]. TNBC does not respond to anticancer drugs that target hormone receptors, such as anastrozole and tamoxifen, or HER2, such as lapatinib and trastuzumab [2]. The current standard treatments for TNBC are surgical resection, radiation therapy, and chemotherapy. Taxanes and anthracyclines, including paclitaxel, docetaxel, doxorubicin, and epirubicin, are chemotherapeutic drugs approved for the treatment of TNBC [4]. To improve the treatment outcomes of TNBC, poly (ADP-ribose) polymerase (PARP) inhibitors, such as talazoparib and olaparib, as well as pembrolizumab, an immune checkpoint inhibitor, can be additionally used in combination [5,6,7]. However, the heterogeneity and complexity of TNBC have led to resistance to clinically approved therapies [8]. The development of drug resistance leads to relapse and therapeutic failure in patients with TNBC [9]. Therefore, it is urgent to find new types of potential anticancer drugs that effectively suppress the proliferation and metastasis of TNBC cells.

Apoptosis evasion is a critical feature of malignant tumor cells [10]. Therefore, the promotion of cancer cell apoptosis has become a central strategy in anticancer therapy. The distinct changes in cellular morphology and biochemical events, including cell shrinkage, chromatin condensation, DNA fragmentation, generation of reactive oxygen species (ROS), and loss of mitochondrial membrane potential, are key features of apoptosis [10,11]. The intrinsic and extrinsic pathways of apoptosis activate caspases, a family of cysteine proteases. Activated initiator caspases, such as caspase-8 and -9, cleave and activate downstream effector caspases, such as caspase-3 and -7, which execute cellular apoptosis by cleaving a variety of substrate proteins that control the cell cycle, DNA repair, and gene expression [12,13]. Therefore, novel compounds capable of activating the apoptotic pathways and central apoptosis mediators in TNBC cells can be considered potential anticancer agents for TNBC treatment. 

The leading cause of death in patients with TNBC is distant metastases, which are particularly likely to recur and spread to the brain and lungs [14,15]. TNBC metastasis is closely related to abnormal induction of epithelial-mesenchymal transition (EMT) [16]. During EMT, the adhesion ability, polarity, and differentiation of epithelial cells decrease; however, their migration and invasion capacities increase [17]. EMT is triggered by several signaling pathways, including transforming growth factor-β, Wnt/β-catenin, Notch, and Janus kinase/signal transducer and activator of transcription 3 (JAK/STAT3) [18,19]. These signaling pathways activate transcription factors such as Slug, Snail, Twist, and ZEB1, which downregulate the expression of the epithelial markers E-cadherin, Claudin-1, and cytokeratin and upregulate the expression of the mesenchymal markers N-cadherin and vimentin [20]. Thus, targeting EMT may be an effective method to prevent TNBC metastasis and recurrence. 

**Figure 1:**
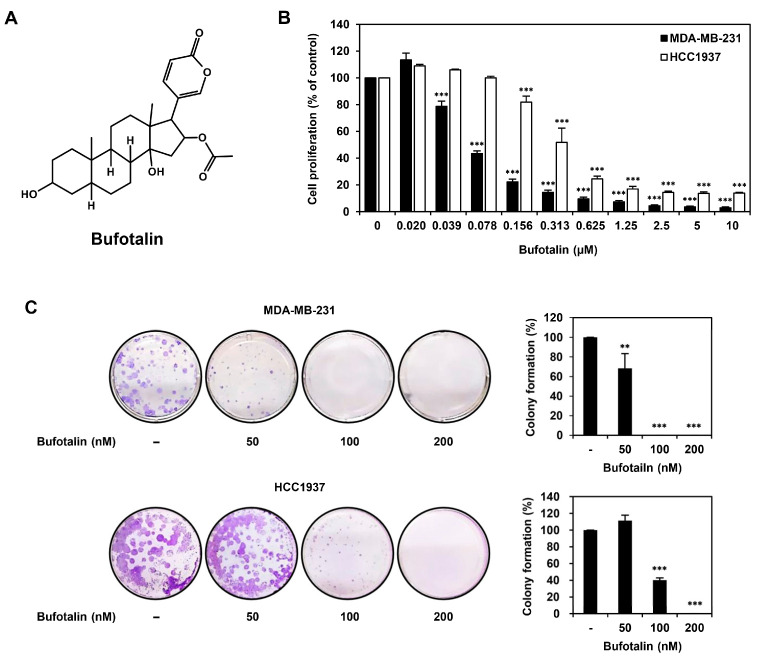
Effect of bufotalin on the proliferation of TNBC cell lines. (**A**) Chemical structure of bufotalin. (**B**) MDA-MB-231 and HCC1937 cells were incubated for 72 h after treatment with bufotalin (0–10 μM). The CellTiter-Glo^®^ luminescent assay was used to measure cell proliferation. (**C**) MDA-MB-231 and HCC1937 cells were incubated for 14 days after treatment with bufotalin (50, 100, 200 nM). Formed cell colonies were stained with a crystal violet solution. ** *p* < 0.01, *** *p* < 0.001 vs. the control.

Bufotalin, a steroid bufadienolide (C_26_H_36_O_6_), is one of the main active ingredients in the traditional Chinese medicine Chan Su, which is a dried secretion from the skin and parotid venom glands of toads (*B. gargarizans*) (Figure 1A) [21]. Chan Su has long been used as a cardiotonic, antibacterial, antiviral, antitumor, analgesic, and local anesthetic agent in China and other Asian countries [21]. Previous studies have shown that bufotalin exhibits antiviral, anti-inflammatory, and anticancer activities [22,23,24]. Bufotalin ameliorates chronic inflammatory autoimmune diseases by inhibiting Th17 polarization and cytokine secretion [23]. Bufotalin also has broad anti-coronavirus activity [24]. The antitumor effects of bufotalin have been demonstrated in various cancer cell lines [25]. Bufotalin promotes apoptosis in the liver cancer cell line Hep3B by activating caspases and apoptosis-inducing factor (AIF) [26]. It also suppresses the proliferation of A375 melanoma cells by causing apoptosis and cell cycle arrest through the inactivation of the serine/threonine kinase AKT [27]. In addition, bufotalin triggers apoptosis mediated by p53 in esophageal squamous cell carcinoma cells and leads to ferroptosis by promoting the degradation of glutathione peroxidase 4 in A549 non-small cell lung cancer cells [28,29]. However, no studies have demonstrated that bufotalin inhibits the proliferation and metastasis of TNBC cells. In the current study, we assessed the antiproliferative and antimetastatic effects of bufotalin on TNBC cells for the first time. In addition, it was confirmed that the anticancer activity of bufotalin against TNBC cells was related to the regulation of the apoptosis pathway, EMT, and STAT3 signaling.

## 2. Results

### 2.1. Bufotalin Inhibits TNBC Cell Proliferation

To investigate whether bufotalin affects the proliferation of TNBC cells, the human MDA-MB-231 and HCC1937 TNBC cell lines were treated with bufotalin at the indicated doses (0–10 μM) for 72 h. Cell proliferation was then measured using a luminescent adenosine triphosphate (ATP) detection assay. Bufotalin dose-dependently inhibited the proliferation of MDA-MB-231 and HCC1937 TNBC cells with 78 and 370 nM of the IC_50_ value, respectively (Figure 1B). Next, we evaluated the effect of bufotalin on the colony-forming ability of MDA-MB-231 and HCC1937 cells. Bufotalin treatment dose-dependently suppressed clonogenic growth of both TNBC cell lines (Figure 1C). These results demonstrate that bufotalin inhibits the proliferation of TNBC cells.

### 2.2. Bufotalin Induces Cell Cycle Arrest and Apoptosis in TNBC Cells

**Figure 2:**
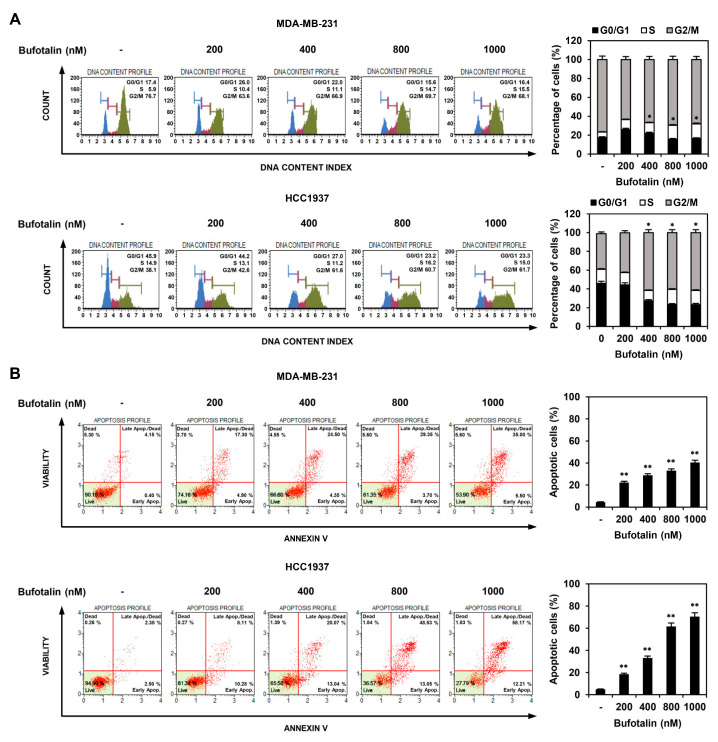
Effect of bufotalin on the cell cycle distribution and apoptotic cell death in TNBC cell lines. (**A**,**B**) MDA-MB-231 and HCC1937 cells were incubated for 72 h after treatment with bufotalin (200, 400, 800, 1000 nM). (**A**) Phases of the cell cycle were measured by the Muse Cell Analyzer after staining the TNBC cells with a Muse^®^ Cell Cycle reagent. (**B**) Apoptotic cells were measured by the Muse Cell Analyzer after staining the TNBC cells with a Muse^®^ Annexin V and Dead Cell reagent. * *p* < 0.05, ** *p* < 0.01 vs. the control.

To determine whether bufotalin inhibited TNBC cell proliferation through regulation of the cell cycle and apoptosis, we first investigated the effect of bufotalin on cell cycle distribution using flow cytometry. MDA-MB-231 and HCC1937 TNBC cell lines were treated with 200, 400, 800, and 1000 nM of bufotalin for 72 h. Compared to the untreated cells, treatment with bufotalin increased the S and G2/M phase cell populations in MDA-MB-231 and HCC1937 cells, respectively, in a dose-dependent manner (Figure 2A). These results indicate that bufotalin inhibited TNBC cell proliferation by inducing cell cycle arrest at the S phase in MDA-MB-231 cells and the G2/M phase in HCC1937 cells. The effect of bufotalin on cellular apoptosis was analyzed using flow cytometry. Bufotalin treatment for 72 h increased the proportion of apoptotic MDA-MB-231 and HCC1937 cells compared to that in untreated control cells in a dose-dependent manner (Figure 2B). Thus, the anti-proliferative effect of bufotalin on TNBC cells may be related to cell cycle arrest and induction of apoptosis. 

### 2.3. Bufotalin Activates Caspase-Mediated Apoptotic Pathway in TNBC Cells

**Figure 3:**
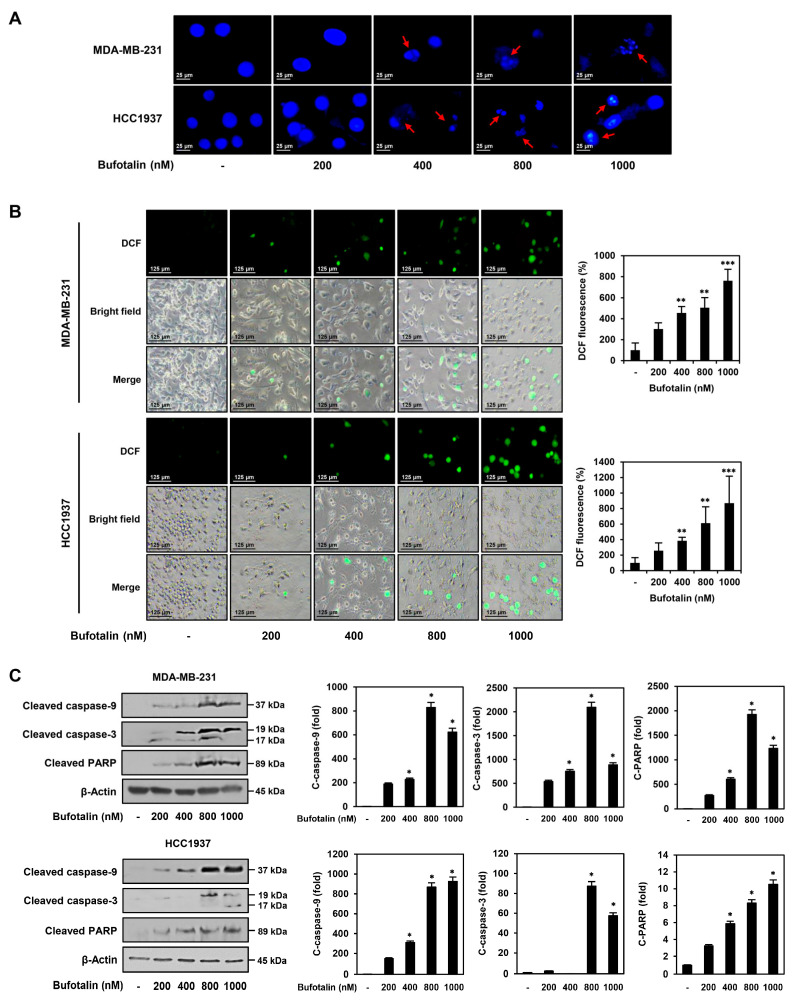
Effect of bufotalin on the apoptotic characteristics of TNBC cell lines. (**A**–**C**) MDA-MB-231 and HCC1937 cells were incubated for 48 h after treatment with bufotalin (200, 400, 800, 1000 nM). (**A**) Cell nuclei were stained with DAPI, and nuclear morphology was observed using a fluorescence microscope. The condensed and fragmented nuclei of cells are marked by red arrows. (**B**) Intracellular ROS produced were observed after staining with DCFH-DA under a fluorescence microscope. To quantify the ROS levels, the intensity of DCF fluorescence was measured by densitometry. (**C**) Protein expression levels of the apoptosis regulators were measured by Western blotting. The levels of β-actin were used as a loading control, and band intensity was measured by densitometry for quantification. * *p* < 0.05, ** *p* < 0.01, *** *p* < 0.001 vs. the control.

To further verify the TNBC cell apoptosis induced by bufotalin, we first investigated whether bufotalin causes nuclear morphological changes in MDA-MB-231 and HCC1937 TNBC cell lines. Staining of nuclei with 4′,6-diamidino-2-phenylindole (DAPI) showed that bufotalin treatment for 48 h induced nuclear condensation and fragmentation in both the cell lines (Figure 3A). Next, to determine whether bufotalin affects the generation of ROS, which plays a central role in inducing apoptosis, intracellular ROS levels were measured using the fluorogenic indicator dichloro-dihydro-fluorescein diacetate (DCFH-DA). Bufotalin treatment for 48 h significantly increased ROS generation in MDA-MB-231 and HCC1937 cells in a dose-dependent manner (Figure 3B). We further evaluated the effect of bufotalin on the expression of caspases, which are key mediators of apoptosis and are activated by proteolytic cleavage. Active caspase-9 triggers cleavage and activation of downstream caspase-3. Subsequently, caspase-3 promotes apoptosis by cleaving the DNA repair enzyme PARP [30]. Western blot analysis revealed that bufotalin treatment for 48 h increased the expression levels of cleaved caspase-9, caspase-3, and PARP in MDA-MB-231 and HCC1937 cells (Figure 3C). These results suggest that bufotalin induces apoptosis by activating the caspase-mediated apoptotic pathway in TNBC cells.

### 2.4. Bufotalin Suppresses TNBC Cell Migration and Invasion

**Figure 4:**
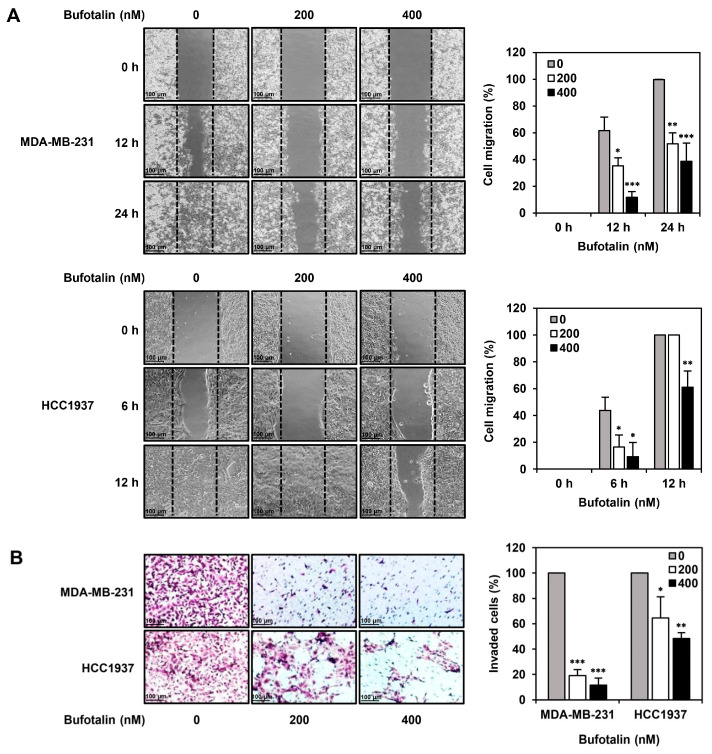
Effect of bufotalin on the metastatic ability of TNBC cell lines. (**A**) Cell migration was evaluated by wound healing assay. After treatment with bufotalin (200, 400 nM), MDA-MB-231 and HCC1937 cells that migrated into the gap were observed at the indicated time points under an optical microscope. The gap area was measured to quantify cell migration. The boundaries of the gap at 0 h are marked with black dashed lines. (**B**) Cell invasion was evaluated using the transwell chamber containing membrane inserts (8.0 μm pore) coated with Matrigel. MDA-MB-231 and HCC1937 cells were incubated for 24 h after treatment with bufotalin (200, 400 nM). The invaded cells were stained with H&E and counted using an optical microscope. * *p* < 0.05, ** *p* < 0.01, *** *p* < 0.001 vs. the control.

In addition to the antiproliferative activity of bufotalin against TNBC cells, we investigated whether bufotalin could inhibit the metastatic ability of TNBC cells. A wound-healing assay was employed to measure TNBC cell migration. Wound closure by MDA-MB-231 and HCC1937 TNBC cell lines was observed after 24 and 12 h of incubation, respectively. Bufotalin treatment significantly reduced the migration of both TNBC cell lines compared to that of the untreated cells (Figure 4A). Next, the effect of bufotalin on the invasion ability of TNBC cells was evaluated using a transwell chamber system coated with Matrigel matrix. Bufotalin treatment for 24 h inhibited the invasiveness of MDA-MB-231 and HCC1937 TNBC cell lines in a dose-dependent manner (Figure 4B). These results suggest that bufotalin effectively suppresses TNBC metastasis.

### 2.5. Bufotalin Modulates Major Molecular Markers Involved in TNBC Metastasis

**Figure 5:**
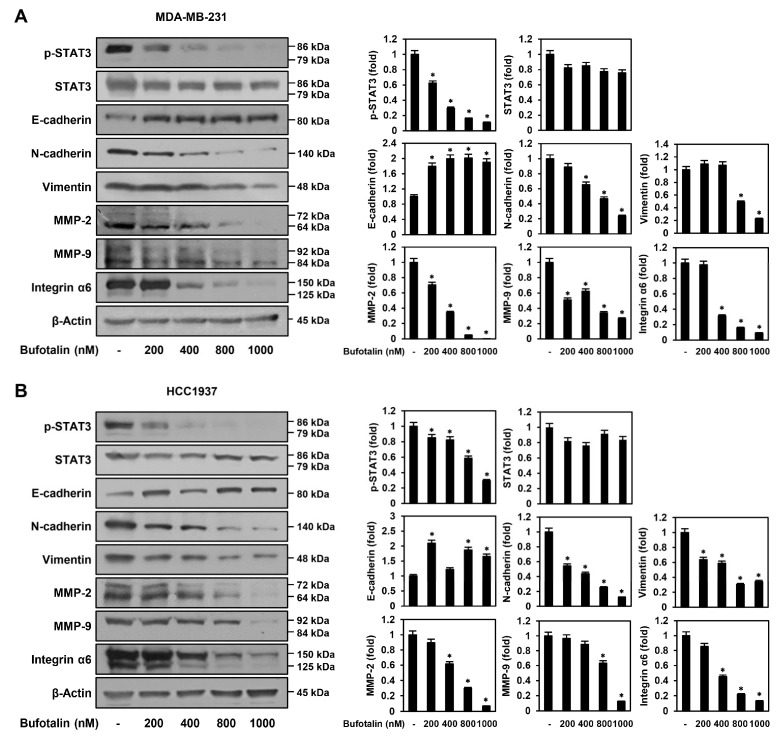
Effect of bufotalin on the major molecular markers involved in TNBC cell metastasis. (**A**) MDA-MB-231 and (**B**) HCC1937 cells were incubated for 24 h after treatment with bufotalin (200, 400, 800, 1000 nM). Protein expression levels were measured by Western blotting. The levels of β-actin were used as a loading control, and band intensity was measured by densitometry for quantification. * *p* < 0.05 vs. the control.

To determine the molecular mechanism by which bufotalin suppresses the metastasis of TNBC cells, we assessed whether bufotalin affects the expression of key molecular markers, including EMT regulators, matrix metalloproteinases (MMPs), and integrin α6, which play important roles in TNBC metastasis [16,31,32]. After bufotalin treatment of MDA-MB-231 and HCC1937 cells, expression of the epithelial cell-specific marker E-cadherin was upregulated, whereas expression of the mesenchymal cell-specific markers N-cadherin and vimentin was downregulated (Figure 5). In addition, bufotalin significantly reduced the expression of MMP-2, MMP-9, and integrin α6 in both TNBC cell lines (Figure 5). These data indicate that the anti-metastatic activity of bufotalin in TNBC cells may be associated with the downregulation of EMT, MMPs, and integrins that contribute to TNBC cell migration and invasion.

Aberrant STAT3 signaling is a crucial driver of TNBC proliferation, metastasis, and chemoresistance [18]. Therefore, we examined whether bufotalin regulates the activation of STAT3 in TNBC cells. Bufotalin potently inhibited the phosphorylation of STAT3 in MDA-MB-231 and HCC1937 cells without significantly inhibiting the total protein levels of STAT3 (Figure 5). In addition, colivelin, a STAT3 activator, partially restored the inhibitory effects of bufotalin on STAT3 phosphorylation and cell viability in both TNBC cell lines (Supplementary Figures S1 and S2). These results suggest that bufotalin may exhibit anticancer activity against TNBC cells via the downregulation of the STAT3 signaling pathway.

## 3. Discussion

TNBC is a highly aggressive and metastatic subtype of breast cancer with high mortality rates and no effective targeted therapy [2,3]. Several chemotherapeutic drugs, such as taxanes and anthracyclines, in combination with PARP and immune checkpoint inhibitors, are available for the treatment of TNBC; however, they exhibit low efficacy and side effects [4,5,6,7]. Therefore, the search for novel anticancer agents is urgently required to achieve effective chemotherapy for TNBC. 

Bufadienolides, including telocinobufagin, bufotalin, bufalin, cinobufotalin, and cinobufagin, have been isolated and identified in traditional Chinese medicine Chan Su (dried toad skin secretions) [33]. Accumulating evidence has revealed the antitumor activity of bufadienolides in several cancer types. Multiple studies have focused on the antitumor effects of bufadienolides in breast cancer cells [25]. Bufalin suppressed the growth of the human breast cancer cell lines MCF-7 and MDA-MB-231 by triggering necroptosis through the ROS-mediated receptor-interacting protein (RIP)1/RIP3/PARP-1 pathways [34]. Moreover, bufalin significantly promoted TNF-related apoptosis-inducing ligand (TRAIL)-induced apoptosis in MCF-7 and MDA-MB-231 cells by downregulating the STAT3/myeloid cell leukemia-1 pathway [35]. Cinobufotalin exhibited anticancer activity in MCF-7 cells by modulating the expression of the non-receptor tyrosine kinase SRC and the cyclin-dependent kinase inhibitor 2A [36]. Cinobufagin induces apoptosis and G1 phase arrest in MCF-7 cells by affecting Bax and Bcl-2 expression [37]. Telocinobufagin inhibited the migration and invasion of 4T1 murine breast cancer cells by repressing EMT through downregulation of the phosphoinositide-3-kinase (PI3K)/AKT/extracellular signal-regulated kinase (ERK)/Snail pathway [38]. However, the anticancer effect and underlying molecular mechanisms of action of bufotalin in TNBC cells have not yet been fully elucidated. In the current study, we demonstrated, for the first time, the antiproliferative and antimetastatic activities of bufotalin against TNBC cells through the regulation of the apoptotic pathway, EMT, and STAT3 signaling. 

Our results showed that bufotalin potently inhibited TNBC cell proliferation by inducing cell cycle arrest and apoptosis. Bufotalin arrested the cell cycle in the S and G2/M phases in the MDA-MB-231 and HCC1937 cell lines, respectively. It also promoted apoptotic cell death by activating key regulatory mechanisms of apoptosis, including nuclear fragmentation, increased ROS production, and activation of the caspase cascade in both TNBC cell lines. Bufotalin effectively suppressed the migration and invasion of MDA-MB-231 and HCC1937 cells. Notably, the anti-metastatic activity of bufotalin in TNBC cells is related to the downregulation of EMT, MMPs, and integrin α6. EMT is an important step in TNBC metastasis [16]. Bufotalin reverses EMT by augmenting the expression of the epithelial cell marker E-cadherin and reducing the expression of the mesenchymal cell markers N-cadherin and vimentin in MDA-MB-231 and HCC1937 cells. MMPs are a family of zinc-dependent proteases that degrade the extracellular matrix (ECM) and play important roles in TNBC metastasis [32]. Bufotalin decreased the expression of MMP-2 and MMP-9, the major MMPs that are highly expressed in TNBC. Integrins are transmembrane receptors that play important roles in mediating cell-cell and cell-ECM adhesion and regulate diverse cellular responses, including cell survival, proliferation, migration, and invasion [39]. In addition, the aberrant expression of integrins, including α6 and β1, is closely linked to the invasiveness and metastasis of cancer cells, including breast cancer [39]. Bufotalin markedly inhibited integrin α6 expression in both TNBC cell lines. Therefore, our data demonstrate the antiproliferative and antimetastatic activities of bufotalin in TNBC cells and identify the anticancer molecular mechanisms involved in the regulation of caspase-mediated apoptosis, EMT, MMP, and integrins. 

**Figure 6:**
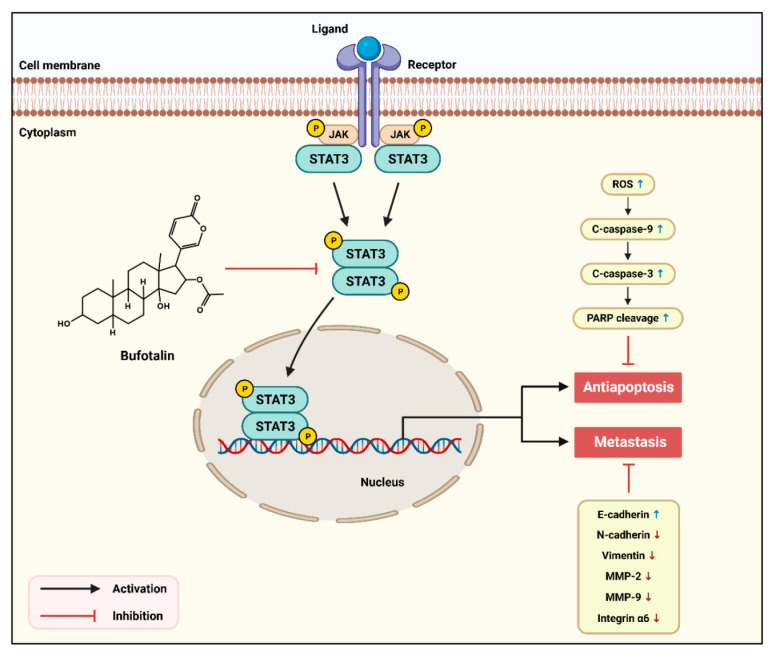
Proposed molecular mechanism of anticancer action of bufotalin in TNBC cells.

Recent studies demonstrated that STAT3 plays a pivotal role in TNBC growth and metastasis [18]. STAT3 acts as a transcriptional activator that regulates multiple target oncogenes and promotes the proliferation, anti-apoptosis, angiogenesis, metastasis, and chemoresistance of cancer cells [40,41]. It has been reported that STAT3-mediated breast cancer cell metastasis is associated with the upregulation of vimentin, MMP-2, and MMP-9 [42]. Inhibition of STAT3 signaling also reduces the expression of EMT inducers and MMPs in breast cancer cells [43]. STAT3 activation promotes integrin-mediated cell adhesion and intracellular signaling in breast cancer cells [44]. Thus, STAT3 may be an upstream regulator contributing to the activation of apoptotic evasion, EMT, MMPs, and integrins in TNBC cells. Our results showed that bufotalin significantly downregulated STAT3 signaling in both the MDA-MB-231 and HCC1937 cell lines, suggesting that bufotalin suppresses TNBC cell proliferation and metastasis by blocking the STAT3 signaling pathway (Figure 6). 

Collectively, our findings suggest that bufotalin is a promising anticancer agent that effectively suppresses TNBC cell growth and metastasis. Recently, Chan Su was approved by the Chinese FDA as injections, capsules, oral solutions, and tablets [25]. Clinically, Chan Su is used for the treatment of severe upper and lower respiratory tract infections, chronic hepatitis B, and several cancer types, including liver, prostate, and colorectal cancers [45,46]. However, the clinical efficacy and safety of the components of Chan Su, including bufotalin, remain unclear. Therefore, further in vivo studies are required to definitively verify the efficacy and safety of bufotalin for the treatment of TNBC.

## 4. Materials and Methods

### 4.1. Materials

Bufotalin was obtained from MedChemExpress (South Brunswick, NJ, USA). DMEM, RPMI-1640, and trypsin were obtained from HyClone (Marlborough, MA, USA). Fetal bovine serum (FBS) and penicillin/streptomycin/amphotericin B solution were obtained from R&D Systems (Minneapolis, MN, USA) and Lonza (Walkersville, MD, USA), respectively. Dichlorodihydrofluorescein diacetate (DCFH-DA), crystal violet, eosin, hematoxylin, gelatin, and 4’,6-diamidine-2’-phenylindole dihydrochloride (DAPI) were obtained from Sigma-Aldrich (St. Louis, MO, USA). Ibidi culture inserts and Transwell chamber inserts were obtained from ibidi GmbH (Munich, Germany) and SPL Life Sciences (Pocheon, Republic of Korea), respectively. Matrigel and polyvinylidene difluoride (PVDF) membranes were obtained from Corning (Tewksbury, MA, USA) and EMD Millipore (Hayward, CA, USA), respectively. Antibodies for detecting phospho-STAT3 (Tyr705, #9145), STAT3 (#9139), cleaved PARP (#9542), MMP-2 (#4022), MMP-9 (#3852), cleaved caspase-3 (#9661), cleaved caspase-9 (#9501), integrin α6 (#3750), β-actin (#4967), mouse IgG (#7076), and rabbit IgG (#7074) were obtained from Cell Signaling Technology (Danvers, MA, USA). Antibodies against vimentin (#A11952), N-cadherin (#A0433), and E-cadherin (#A11492) were obtained from ABclonal (Woburn, MA, USA). An enhanced chemiluminescence (ECL) kit was obtained from Bio-Rad Laboratories (Hercules, CA, USA).

### 4.2. Cell Culture

Human TNBC cell lines MDA-MB-231 (KCLB No. 30026) and HCC1937 (KCLB No. 9S1937) were provided by the Korean Cell Line Bank (Seoul, Republic of Korea). Each cell line was identified by STR analysis. MDA-MB-231 and HCC1937 cells were grown in DMEM and RPMI-1640 media containing 10% FBS and 1% penicillin/streptomycin/amphotericin B, respectively. The TNBC cell lines were incubated at 37 °C in a humidified CO_2_ incubator with 5% CO_2_ (Thermo Scientific, Vantaa, Finland). 

### 4.3. Cell Proliferation Assay

MDA-MB-231 and HCC1937 cells were inoculated at a density of 3 × 10^3^ cells per well in 96-white-well culture plates. The TNBC cells were incubated for 72 h after treatment with bufotalin (0–10 μM). To measure cell proliferation, the CellTiter-Glo^®^ 2.0 Cell Viability Assay (Promega, Madison, WI, USA) was performed according to the manufacturer’s instructions [47]. Luminescence was measured using a BioTek multimode plate reader (Winooski, VT, USA). Calculation of IC_50_ was performed with GraphPad Prism 6 software (La Jolla, CA, USA).

### 4.4. Colony Formation Assay

MDA-MB-231 and HCC1937 cells were inoculated at a density of 1 × 10^3^ cells per well in six-well culture plates. The TNBC cells were incubated for 14 days after treatment with bufotalin (50, 100, 200 nM). Formed cell colonies were fixed using a 3.7% formaldehyde solution and then washed with phosphate-buffered saline (PBS). After staining with 0.5% crystal violet solution for 15 min, visible cell colonies were observed and counted. 

### 4.5. Cell Cycle Analysis

MDA-MB-231 and HCC1937 cells were inoculated at a density of 1 × 10^5^ cells per well in 6-well culture plates. The TNBC cells were incubated for 72 h after treatment with bufotalin (200, 400, 800, 1000 nM). The cells were collected and then fixed using 70% ethanol at −20 °C for 3 h. The TNBC cells were washed with PBS and then stained by adding 200 µL of Muse^®^ Cell Cycle reagent (Luminex, Austin, TX, USA) according to the manufacturer’s instructions [47]. The cell cycle profile of each sample was measured with the Guava^®^ Muse^®^ Cell Analyzer with MuseSoft_V1.8.0.3 (Luminex, Austin, TX, USA).

### 4.6. Analysis of Apoptotic Cell Death

MDA-MB-231 and HCC1937 cells were inoculated at a density of 1 × 10^5^ cells per well in six-well culture plates. The TNBC cells were incubated for 72 h after treatment with bufotalin (200, 400, 800, 1000 nM). The cells were collected and then stained by adding 100 μL of Muse^®^ Annexin V & Dead Cell reagent (Luminex, Austin, TX, USA) according to the manufacturer’s instructions [47]. The percentage of apoptotic cells was determined using the Guava^®^ Muse^®^ Cell Analyzer with MuseSoft_V1.8.0.3 (Luminex, Austin, TX, USA).

### 4.7. Analysis of Nuclear Morphology

MDA-MB-231 and HCC1937 cells were inoculated at a density of 5 × 10^4^ cells per well in 24-well culture plates. The TNBC cells were incubated for 48 h after treatment with bufotalin (200, 400, 800, 1000 nM). After washing with PBS, the cells were treated with 20 μg/mL of DAPI and incubated for 1 h [48]. The stained cellular nuclei were observed using the Optinity KI-2000F fluorescence microscope (Korea Lab Tech, Seong Nam, Republic of Korea).

### 4.8. ROS Generation Analysis

MDA-MB-231 and HCC1937 cells were inoculated at a density of 5 × 10^4^ cells per well in 24-well culture plates. The TNBC cells were incubated for 48 h after treatment with bufotalin (200, 400, 800, 1000 nM). The cells were washed with PBS and then treated with 20 μM of DCFH-DA [48]. After incubation for 20 min, intracellular ROS produced were detected using the Optinity KI-2000F fluorescence microscope (Korea Lab Tech, Seong Nam, Republic of Korea). To quantify the ROS levels, the intensity of DCF fluorescence was measured with the ImageJ 1.5 software program from NIH (Bethesda, MD, USA). The DCF fluorescence levels of untreated control cells were normalized to 100%.

### 4.9. Wound Healing Assay

After attaching ibidi culture inserts to each well, MDA-MB-231 and HCC1937 cells were inoculated at a density of 5 × 10^4^ cells per insert in 24-well culture plates and then incubated for 24 h. The inserts were removed, and the cells were treated with bufotalin (200, 400 nM) for 12 or 24 h. The images of migrated cells were obtained at each time point (0, 6, 12, 24 h) using the Olympus optical microscope (Tokyo, Japan) at a magnification of 200×. The area of the gap was measured, and the cell migration results were expressed as a percentage of control.

### 4.10. Invasion Assay

The transwell chamber containing polycarbonate membrane inserts with 8.0 μm pore size was used to measure TNBC cell invasiveness [49]. The outside of the membrane insert was coated with 10 μL of gelatin (1 mg/mL) and dried at room temperature for 1 h. The inside of the membrane insert was then coated with 10 μL of Matrigel (3 mg/mL) and dried at room temperature for 1 h. MDA-MB-231 and HCC1937 cells were inoculated at a density of 5 × 10^4^ cells per well in the upper chamber of the membrane insert, and bufotalin (200, 400 nM) was added to the lower chamber. After incubation for 24 h, the invaded cells were fixed using 70% methanol and then stained using hematoxylin and eosin (H&E). The images of invaded cells were taken using the Olympus optical microscope (Tokyo, Japan) at a magnification of 200×. The total invaded cells were counted, and cell invasion results were expressed as a percentage of control.

### 4.11. Western Blotting

Sodium dodecyl sulfate-polyacrylamide gel electrophoresis (SDS-PAGE) was performed to separate TNBC cell lysate samples prepared at equal protein concentrations according to molecular weight. The proteins separated on the gel were then transferred onto the PVDF membrane (pore size 0.2 μm) by electroblotting. To block the non-specific binding of antibodies, the transferred blots were incubated with 5% non-fat milk dissolved in Tris-buffered saline containing 1 × Tween-20 (TBST) for 1 h at room temperature with gentle agitation. For immunolabeling, the blots were incubated with specific primary antibodies (dilution 1:2000–1:10,000) at 4 °C overnight with gentle agitation. Thereafter, the membranes were washed thrice using TBST and then incubated with horseradish peroxidase (HRP)-conjugated secondary antibodies (dilution 1:3000) for 1 h at room temperature with gentle agitation. Subsequently, immunolabeling was detected by an ECL kit according to the manufacturer’s instructions [47]. The results were quantified by calculating the band intensity of the target protein relative to β-actin using the ImageJ 1.5 software program from NIH (Bethesda, MD, USA). The expression ratio of each target protein to β-actin in untreated control cells was normalized to onefold.

### 4.12. Statistical Analysis

Statistical analyses were done with ANOVA followed by Tukey’s post-hoc test using SPSS version 9.0 software (Chicago, IL, USA). A *p* < 0.05 was considered to indicate a statistically significant difference. Data are presented as the mean ± standard deviation (SD).

## 5. Conclusions

In summary, bufotalin suppressed the growth and metastasis of MDA-MB-231 and HCC1937 TNBC cell lines. Bufotalin induced cell cycle arrest and apoptosis and inhibited cell migration and invasion in both TNBC cells. Furthermore, our results demonstrated that the anticancer activity of bufotalin against TNBC cells was related to the regulation of the caspase-mediated apoptotic pathway, EMT, MMP, integrin, and STAT3 signaling. Therefore, bufotalin has the chemotherapeutic potential to effectively suppress TNBC.

## Data Availability

The data that support the findings of this study are available from the corresponding author upon reasonable request.

## Supplementary Materials

The following supporting information can be downloaded at: https://www.mdpi.com/article/10.3390/molecules28196783/s1. Figure S1. Colivelin partially restores the inhibitory effect of bufotalin on STAT3 phosphorylation in TNBC cell lines; Figure S2. Colivelin partially restores the inhibitory effect of bufotalin on cell viability in TNBC cell lines.
