# Supplementary material for: Bufotalin Suppresses Proliferation and Metastasis of Triple-Negative Breast Cancer Cells by Promoting Apoptosis and Inhibiting the STAT3/EMT Axis
Source: Molecules. 2023 Sep 23;28(19):6783. doi: 10.3390/molecules28196783 (PMC10574664; doi:10.3390/molecules28196783)
Supplement: Supplementary file 1 [file molecules-28-06783-s001.zip › molecules-2569019-supplementary.pdf]

Article

Bufotalin Suppresses Proliferation and Metastasis of Triple-Negative Breast Cancer Cells by Promoting Apoptosis and Inhibiting the STAT3/EMT Axis

So Jin Park [1] and Hye Jin Jung [1,2,3,*]

[1] Department of Life Science and Biochemical Engineering, Graduate School, Sun Moon University, Asan 31460, Republic of Korea; psj1867@naver.com
[2] Department of Pharmaceutical Engineering and Biotechnology, Sun Moon University, Asan 31460, Republic of Korea
[3] Genome-Based BioIT Convergence Institute, Sun Moon University, Asan 31460, Republic of Korea
* Correspondence: poka96@sunmoon.ac.kr; Tel.: +82-41-530-2354; Fax: +82-41-530-2939

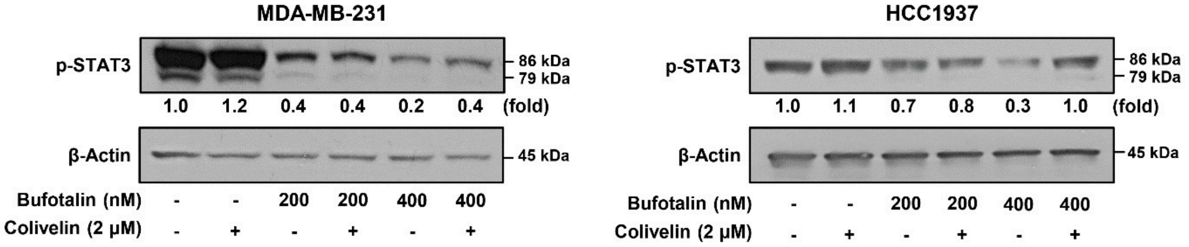

Figure S1. Colivelin partially restores the inhibitory effect of bufotalin on STAT3 phosphorylation in TNBC cell lines. MDA-MB-231 and HCC1937 cells were incubated for 24 h after treatment with colivelin and bufotalin. Protein expression levels were measured by Western blotting. The levels of β-actin were used as a loading control and band intensity was measured by densitometry. The expression ratio of p-STAT3 to β-actin in untreated control cells was normalized to onefold.

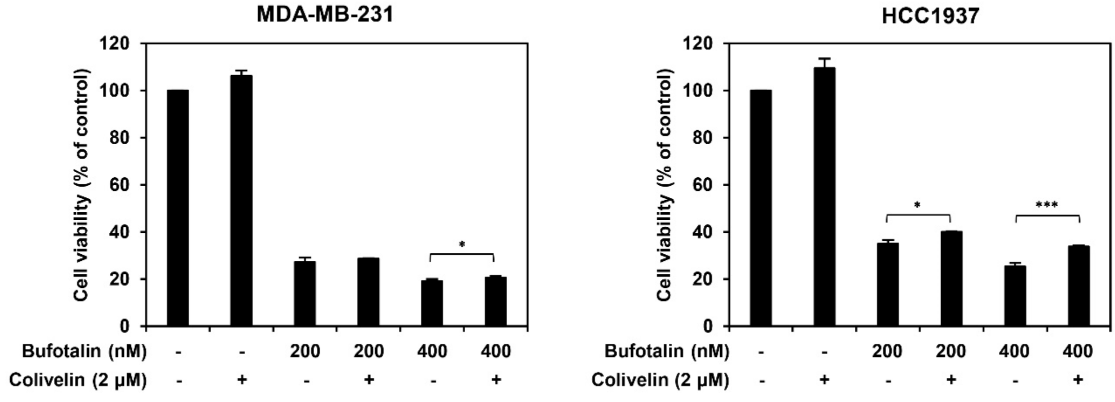

Figure S2. Colivelin partially restores the inhibitory effect of bufotalin on cell viability in TNBC cell lines. MDA-MB-231 and HCC1937 cells were incubated for 48 h after treatment with colivelin and bufotalin. The CellTiter-Glo® luminescent assay was used to measure cell viability. * $p < 0.05$, *** $p < 0.001$.
